# LIPS and PaO_2_/FiO_2_ Combined Plasma Biomarkers Predict Onset of Acute Respiratory Distress Syndrome in Patients of High Risks in SICU: A Prospective Exploratory Study

**DOI:** 10.1155/2024/4936265

**Published:** 2024-09-17

**Authors:** Ziyuan Shen, Zhongnan Yin, Senhao Wei, Zhukai Cong, Feng Zhao, Hua Zhang, Xi Zhu

**Affiliations:** ^1^ Department of Critical Care Medicine Peking University Third Hospital, Beijing 100191, China; ^2^ Biobank Peking University Third Hospital, Beijing 100191, China; ^3^ Center of Epidemiology Peking University Third Hospital, Beijing 100191, China

## Abstract

**Objective:**

To explore and validate the value of clinical parameters combined with plasma biomarkers for predicting acute respiratory distress syndrome (ARDS) in patients of high risks in the surgical intensive care unit (SICU).

**Materials and Methods:**

We conducted a prospective, observational study from January 2020 to December 2023, which enrolled 263 patients of high risks in the SICU of Peking University Third Hospital consecutively; they were classified as ARDS and non-ARDS according to whether ARDS occurred after enrollment. Collected clinical characteristics and blood samples within 24 hr of admission to SICU. Blood samples from the first day to the seventh day of SICU were collected from patients without ARDS, and patients with ARDS were collected until 1 day after ARDS onset, forming data based on time series. ELISA and CBA were used to measure plasma biomarkers. Endpoint of the study was the onset of ARDS. Cox proportional hazard regression analysis was used to find independent risk factors of the onset of ARDS, then constructed a nomogram and tested its goodness-of-fit.

**Results:**

About 84 of 263 patients ended with ARDS. Univariate analysis found 15 risk factors showed differences between ARDS and non-ARDS, namely, interleukin 6, interleukin 8 (IL-8), angiopoietin Ⅱ, LIPS, APACHEⅡ, SOFA, PaO_2_/FiO_2_, age, sex, shock, sepsis, acute abdomen, pulmonary contusion, pneumonia, hepatic dysfunction. We included factors with *p*  < 0.2 in multivariate analysis and showed LIPS, PaO_2_/FiO_2_, IL-8, and receptor for advanced glycation end-products (RAGE) of the first day were independent risk factors for ARDS in SICU, a model combining them was good in predicting ARDS (C-index was 0.864 in total patients of high risks). The median of the C-index was 0.865, showed by fivefold cross-validation in the train cohort or validation cohort. The calibration curve shows an agreement between the probability of predicting ARDS and the actual probability of occurrence. Decision curve analysis indicated that the model had clinical use value. We constructed a nomogram that had the ability to predict ARDS in patients of high risks in SICU.

**Conclusions:**

LIPS, PaO_2_/FiO_2_, plasma IL-8, and RAGE of the first day were independent risk factors of the onset of ARDS. The predictive ability for ARDS can be greatly improved when combining clinical parameters and plasma biomarkers.

## 1. Introduction

Acute respiratory distress syndrome (ARDS) is a critical illness characterized by bilateral chest radiographical opacities with refractory hypoxemia due to noncardiogenic pulmonary edema [1], which is a common cause of respiratory failure in critically ill patients [2]. A multicenter study showed ARDS represented 10.4% of total ICU admissions and 23.4% of all patients requiring mechanical ventilation [3].Due to the high heterogeneity of ARDS, the lack of specific diagnostic criteria and treatment methods, as well as the rapid progression after diagnosis, the mortality rate remains high, currently ranging from 35% to 46% [3, 4, 5, 6]. And COVID-19 led to ARDS in 15% of cases [7], the outcome seemed to be worsen, ranging between 26% and 61.5% in patients admitted into a critical care setting and ranging between 65.7% and 94% in patients who received mechanical ventilation [8, 9]. Although most ARDS survivors recover normal or near-normal pulmonary function, many remain burdened by functional limitations related to muscle weakness, deconditioning, or cognitive impairment [4]. Therefore, there is an eager demand to screen early ARDS patients among high-risk patients as soon as possible and take preventive measures, attempting to reduce the mortality of ARDS and the therapy cost.

The pathogenesis of ARDS includes multiple overlapping and interacting injury response pathways locally and systemically, such as the activation and dysregulation of inflammation and coagulation [10]. However, the most significant factor is the cytokine storm in lung tissue, resulting from a positive feedback loop between the excessive activation of the immune system and the uncontrolled release of cytokines, which leads to severe damage to lung tissue [11, 12].

More and more evidences suggested biomarkers can assist us in predicting ARDS in patients of high risks [13, 14, 15]. However, due to differences in research design, the results obtained vary, and no consensus has been reached so far. We conducted a prospective observational cohort study by observing the occurrence of ARDS among high-risk patients admitted to the surgical intensive care unit (SICU) daily, combining clinical and laboratory indicators. The aim is to establish a more precise ARDS prediction model through precise clinical diagnosis, clinical indicators, and plasma biomarkers reflecting the pathophysiological changes during the progression of ARDS in order to predict ARDS early in clinical practice. Among them, plasma biomarkers included receptor for advanced glycation end-products (RAGE) and Krebs von den Lungen-6 (KL-6) as indicative of alveolar epithelium damage; angiopoietin Ⅱ (AngⅡ) as a marker of vascular endothelium damage; interferon-*γ* (IFN-*γ*), tumor necrosis factor-*α* (TNF-*α*), interleukin 6 (IL-6), interleukin 8 (IL-8), interleukin 10 (IL-10) as mediators in the inflammatory response. We also measured monocyte chemoattractant protein-1 (MCP-1).

## 2. Materials and Methods

### 2.1. Population

The study was performed in a 20-bed SICU of Peking University Third Hospital (Beijing, China) between January 2020 and December 2023. The study design, performance, and report complied with the standards for Reporting of Diagnostic Accuracy guidelines [16]. Patients enrolled in our study were monitored and treated according to international guidelines. Patients with high risks of ARDS who stayed in the SICU longer than 48 hr were prospectively and consecutively enrolled. High risks consisted of shock, multiple trauma, traumatic brain injury, sepsis, acute abdomen, pulmonary, contusion, pneumonia, high-risk parturients, aspiration, spinal corrective surgery, and cervical spinal cord injury. The exclusion criteria included patients without consent; age < 18 years old; organ or bone marrow transplantation; immunodeficiency diseases; receiving cytotoxic therapy; neutropenia (except due to sepsis); developed ARDS before admission; more than 30% of the missing data.

### 2.2. Clinical Endpoints and Definition

ARDS was diagnosed according to the Berlin Definition in 2012 [1]. The primary endpoint was the onset of ARDS within 7 days after enrollment, which was determined by two experienced clinicians who were blinded to the expression of plasma biomarkers. If there was any objection, a third clinician was invited to assist in the diagnosis. If still not certain, the diagnosis would be made again 4–6 hr later until the diagnosis was clear or excluded.

### 2.3. Clinical Data Extraction

All clinical data were prospectively collected on the basis of case report forms. The baseline characteristics and clinical/laboratory parameters were collected from the electronic medical record system within 24 hr of admission into the SICU, including age, gender, height, weight, mean arterial pressure, heart/respiratory rate, temperature, Glasgow coma score, blood oxygen saturation (SpO_2_), methods of respiratory support, use of vasopressors or continuous renal replacement therapy (CRRT). Risk factors like shock, sepsis, traumatic brain injury, pulmonary contusion, spinal corrective surgery, pneumonia, acute abdomen, etc. Risk adjustment factors such as PH < 7.35, hypoalbuminemia, hepatic dysfunction, diabetes, hypertension, coronary heart disease, chronic kidney disease, etc. Laboratory test results included blood gas analysis, red blood cell hematocrit, white blood cell count, platelet count, blood creatinine, and so on. Body mass index (BMI), lung injury prediction score (LIPS), acute physiology and chronic health evaluation Ⅱ (APACHEⅡ), and sequential organ failure assessment (SOFA) were calculated from the baseline data described above.

### 2.4. Samples Collection

**Figure 1:**
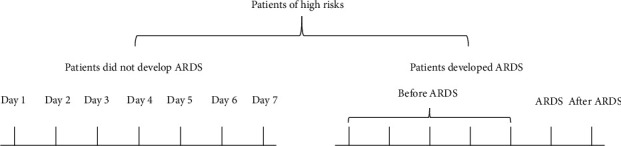
Days of samples collection in patients.

We collected the first clinical parameters and blood samples within 24 hr of admission to SICU. Blood samples of patients who did not develop ARDS were collected from the first day to the seventh day of SICU, and patients who developed ARDS were collected until 1 day after ARDS onset. Days of sample collection are shown in Figure 1.

### 2.5. Biomarkers Measurement

Acquired blood samples were rested for 30 min and subsequently centrifuged at 2,500 rpm at 4°C for 10 min, and supernatant plasma was stored and frozen at −80°C in the Biobank of Peking University Third Hospital until used. Plasma concentrations of IFN-*γ*, TNF-*α*, MCP-1, IL-6, IL-8, and IL-10 were measured by commercial CBA kits (Biolegend, China), and we used ELISA kits (Abebio, China) to measure AngⅡ, KL-6, and RAGE, following the manufacturer's protocol. The biomarkers were measured by technicians of Biobank of Peking University Third Hospital who were blind to clinical data and the physicians in charge were blind to the biomarkers test results.

### 2.6. Statistical Analysis

Plasma biomarkers values underwent logarithmic transformation to achieve approximate normality. Continuous variables were presented as mean ± standard deviation (SD) or median with quartiles 1 and 3 (Q1–Q3) and were compared using the *t*-test or Mann–Whitney *U* test, depending on the results of Kolmogorov–Smirnov test, categorical variables were presented as percentiles and were compared using the *chi*-square test. For all analyses, statistical significance was indicated by two-sided *p*  < 0.05. Cox proportional hazard regression analysis was undertaken to assess the factors associated with the onset of ARDS. The variables with *p*  < 0.2 in the univariate analysis were enrolled in the Cox proportional hazard regression analysis. The contribution of the model to predict ARDS was validated by the net reclassification index (NRI). The Kaplan–Meier survival curve was used to show the probability of ARDS according to a cutoff of the model, index of concordance (C-index), and calibration curve to evaluate the accuracy of the model, decision curve analysis (DCA) to assess the clinical utility, nomogram to facilitate application. The statistical analysis was finished using SPSS 27.0 and R version 4.3.2.

## 3. Results

**Figure 2:**
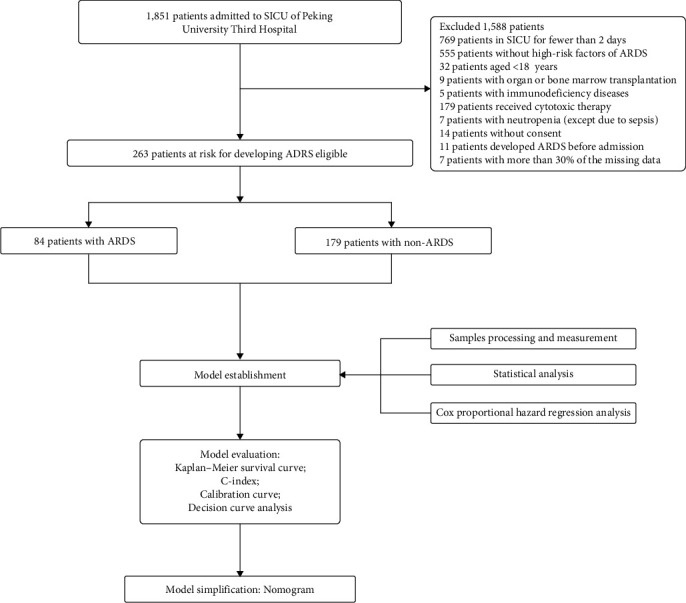
Flowchart of the study selection.

During the study period, 1,851 patients who were admitted to the SICU of Peking University Third Hospital were screened. In total, 769 patients stayed in SICU for less than 2 days; among them, 32 patients aged <18 years old and 555 patients had no high-risk factors of ARDS. After excluding other ineligible patients, 263 patients were finally enrolled, divided into 84 patients with ARDS and 179 patients without ARDS.  Figure 2 shows the process of cohorts selection.

### 3.1. Baseline Characteristics and Clinical/Laboratory Parameters

In our cohort, there were 84 patients ended up with ARDS (there were no patients who developed ARDS on the seventh day). The comparison results showed that there were significant differences between patients with ARDS and without ARDS in age, sex, shock, sepsis, acute abdomen, pulmonary contusion, pneumonia, aspiration, spinal corrective surgery, hepatic dysfunction, use of vasopressors, APACHEⅡ, SOFA, LIPS, PaO_2_/FiO_2_, Days on IMV (invasive mechanical ventilation) and SICU days. Baseline characteristics and clinical/laboratory parameters are shown in Table 1.

### 3.2. Biomarkers in Plasma Samples

In overall enrolled patients, biomarkers in plasma samples of the first day showed that IL-6, IL-8, and AngⅡin ARDS patients were higher than those of patients who would not develop ARDS within 7 days after admission to SICU (*p*  < 0.05), the same was true in patients with extrapulmonary diseases and in moderate, severe ARDS patients. Then, IL-6/IL-10 also showed a significant difference between ARDS and non-ARDS in patients with extrapulmonary diseases and IL-10 in moderate, severe ARDS patients. We also compared the levels of plasma biomarkers between the two groups at other time points, but the results were not significant (shown in Tables S1 and S2).

### 3.3. Features Selected for Predicting ARDS in SICU

**Figure 3:**
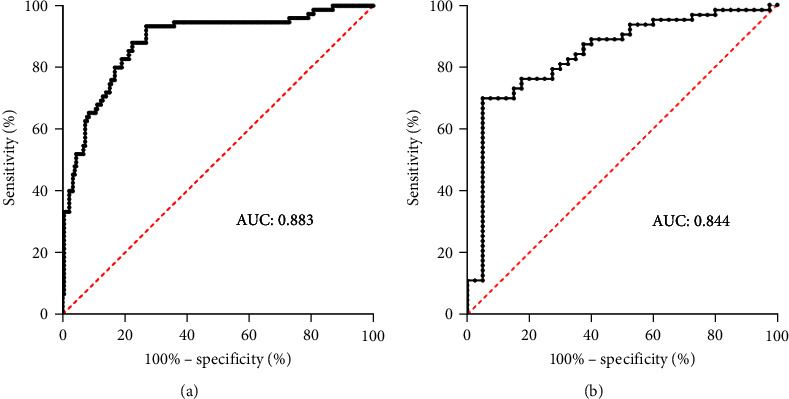
ROC of the model for predicting ARDS in overall patients (a) and sepsis patients (b).

We used Cox proportional hazard regression analysis to build the predictive model. All parameters (except outcome events) in Table 1 were analyzed by univariate analysis. After univariate analysis, 20 variables with *p*  < 0.2 were enrolled in the multivariate analysis. Results indicated that IL-8, RAGE, LIPS, and PaO_2_/FiO_2_ of the first day were independent risk factors for onset of ARDS in SICU patients (details are shown in Table 2); and with variance inflation factor <5, there were no collinearity among all variables (shown in Table S3). C-index of the model when used in overall enrolled patients was 0.864 (95% CI: 0.828−0.900) and when it was used to predict whether a patient would develop ARDS on a specific day, the details of C-index are shown in Table 3. What's more, for all the enrolled patients, the results of the fivefold cross validation are shown in Table 4. Furthermore, the value of model was superior to IL-8, RAGE, LIPS, or PaO_2_/FiO_2_ alone for predicting ARDS, which was supported by NRI (shown in Table S4). We also used ROC (receiver operating characteristic) to verify predictive value of our model, AUC (area under curve) was 0.883 (95% CI: 0.835−0.931, sensitivity 0.933, specificity 0.730) in overall patients, while AUC was 0.844 (95% CI:0.764−0.924) in sepsis patients (Figure 3). Then we eliminated the effects of age and gender through propensity score matching and conducted another Cox proportional hazard regression analysis. The results showed that IL-8, RAGE, LIPS, and PaO_2_/FiO_2_ of the first day were still independent risk factors for onset of ARDS. HR were 0.447^*∗*^ (95% CI:1.006−2.431), 1.771^*∗∗*^ (95% CI: 1.954−17.673), 0.162^*∗∗*^ (95% CI: 1.062−1.301), and −0.006^*∗∗*^ (95% CI: 0.990−0.997), respectively.

**Figure 4:**
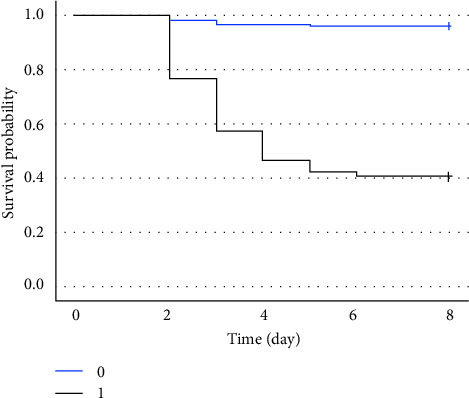
Kaplan–Meier plot of ARDS of patients categorized by model ≥ 3.936 (1) and model < 3.936 (0).

**Figure 5:**
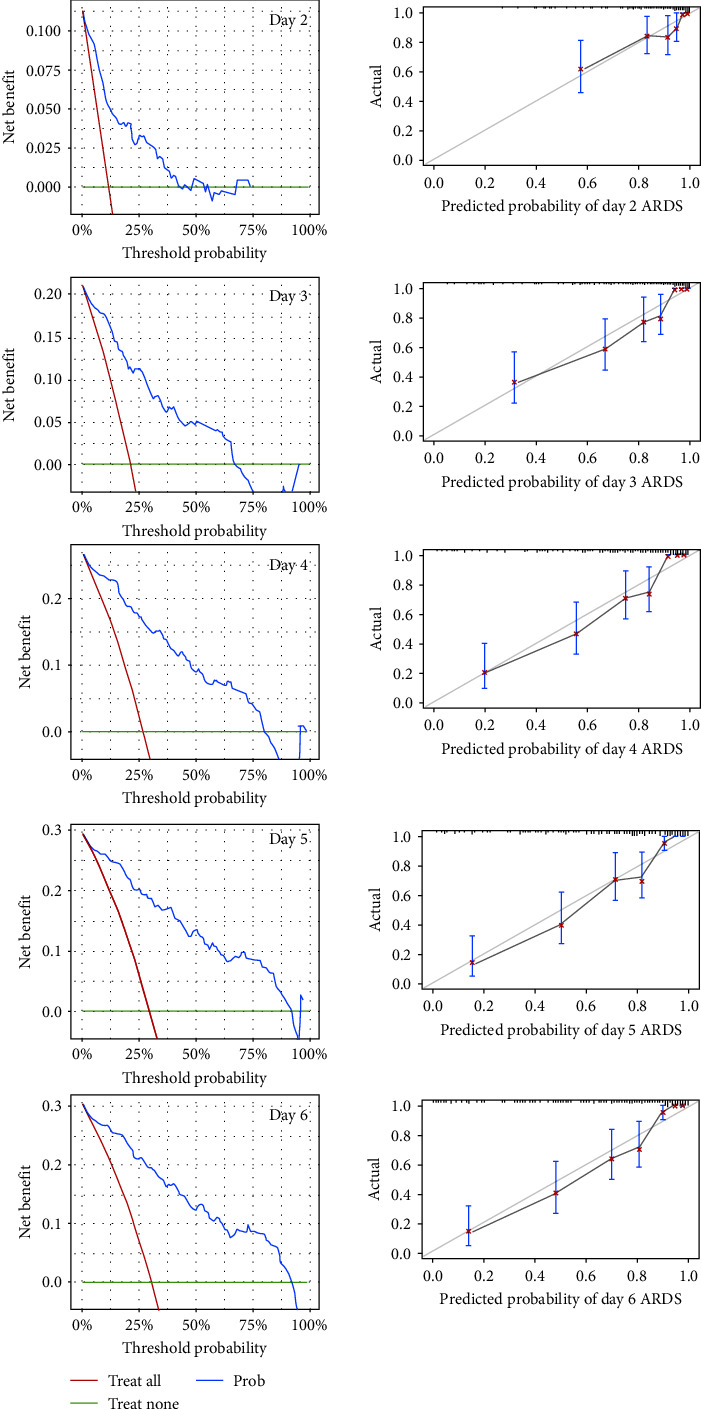
Calibration curve and decision curve analysis of predicting the onset of ARDS on a specific day.

**Figure 6:**
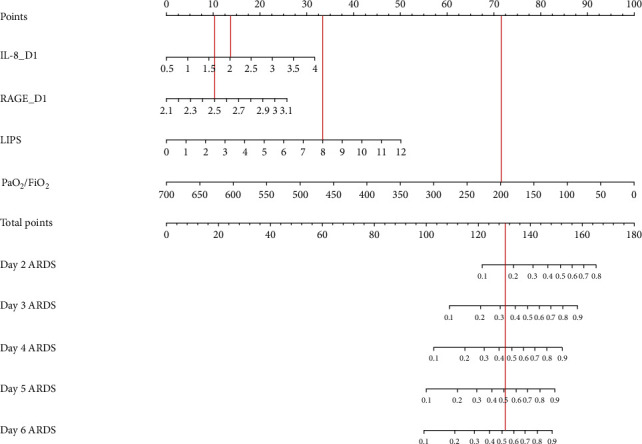
Nomogram model predicting ARDS developed on different days in patients of high risks. The nomogram is used by summing all points identified on the scale for each variable. The total points projected on the bottom scales indicate the probabilities of developing ARDS on the second to the sixth day. IL-8, interleukin-8; RAGE, receptor for advanced glycation end-products; LIPS, lung injury prediction score.

According to the cutoff of the model, we plotted the Kaplan–Meier survival curve, which showed that a high relatively model score was associated with a higher probability of onset of ARDS (Figure 4). Additionally, we drew a calibration curve to evaluate the accuracy of the model and showed that there was good concordance between the predicted and observed values of onset of ARDS, and good clinical application value was reflected by DCA (Figure 5). Plus, a nomogram model that included the important predictors in the Cox analysis was established to predict the onset of ARDS in patients of high risks in SICU (Figure 6).

## 4. Discussion

Our results showed that plasma IL-8, RAGE, LIPS, and PaO_2_/FiO_2_ of the first day were independent risk factors for onset of ARDS, and model established through the probability value (ln [*h* (*t*, *X*)/*h*0 (*t*)] = 0.505×IL-8 + 1.447×RAGE + 0.255×LIPS−0.008×PaO_2_/FiO_2_) was obtained and used to predict ARDS in SICU. The C-index of our model, when used in overall enrolled patients, was 0.864 (95% CI: 0.828−0.900), and 0.937 (95% CI: 0.899−0.975) for predicting ARDS on the second day, 0.923 (95% CI: 0.869−0.977) for the third day, and the predictive value was still superior in the fivefold cross-validation.

IL-8 is one of the neutrophil chemotactic factors, which plays an important role under several pathological and physiological conditions by binding to its cognate G-protein-coupled CXC chemokine receptors, CXCR1 and CXCR2 [17]. A previous study suggested that the elevated plasma levels of IL-8 preceded lung injury in transfusion-related acute lung injury (ALI) [18], and it was more closely correlated with ARDS (OR: 3.21,95% CI: 1.41–7.29) than IL-6 (OR: 2.37, 95% CI: 1.32–4.26), IL-10 (OR: 2.22, 95% CI: 1.14−4.34) and TNF-*α* (OR: 2.45, 95% CI: 1.33−4.51) [19], IL-8 was associated with outcome of ARDS patients [20].

RAGE is a marker of airway epithelial damage, which regulates a variety of important cellular processes, like cell proliferation and migration, inflammation, apoptosis, proliferation, autophagy, and so on [21, 22]. Downs et al. [23] suggested that RAGE plays an important role in the response of alveolar epithelium during the evolution and resolution of lung injury. In the study of macrophages in vitro, S100A12 (RAGE agonist)–RAGE interaction mediated cytokine release and reactive oxygen species (ROS) production [24], and Jabaudon's study showed that the elevated serum RAGE level in ARDS patients could be used as a marker for the diagnosis of ARDS, and was independently related to the death of ARDS patients [25]. A systematic review of biomarkers, including 35 studies, also illustrated RAGE was associated with the onset of ARDS [26]. A combination of RAGE and LIPS was proved to be good at predicting ARDS [27].

In Lorraine's research, the model constructed by plasma surfactant protein-D (SP-D), RAGE, IL-8, club cell secretory protein (CC-16), and IL-6 was helpful for diagnosing the occurrence of ARDS in patients with sepsis. While in trauma patients, a combination of plasma RAGE, procollagen peptide Ⅲ (PCPⅢ), brain-natriuretic peptide (BNP), AngⅡ, IL-10, TNF-*α*, and IL-8 were helpful for diagnosis of ARDS. The variables included in the two models were different, but RAGE and IL-8 were overlapped for diagnosing ARDS in both models. Although the author focused on the diagnostic value of plasma biomarkers for ARDS, rather than prediction, it also provided a basis for our research results [28].

However, AngⅡ, as a marker of vascular endothelium damage, was absent in our prediction model. Researches indicated an upregulation of AngⅡ/AT1R (type 1 angiotensinⅡ receptor)-mediated signaling had been observed, which might be nucleotide phosphodiesterase type 4 (PDE4) [29, 30], while intravenous administration of angiotensin-converting enzyme 2 (ACE2) could reduce inflammatory reaction through downregulation of AT1R [31]. Although AngⅡ showed the difference between ARDS and non-ARDS in baseline parameters, but it was excluded by Cox proportional hazard regression analysis, which might be because it played a greater role between ARDS and non-ARDS (*p*=0.018), who admitted to SICU due to extrapulmonary factors, shown in Table S1(b), but our study built a model based on overall patients. Plus, it was proved that more than 92% of resistance to albumin flux across the alveolar-capillary barrier lied in the epithelial barrier [32, 33]. Injury to the endothelial cells alone is not sufficient to induce pulmonary edema [34]. Thus, alveolar epithelium might play a more important role in the onset of ARDS than vascular endothelium.

KL-6 is a glycoprotein secreted by alveolar typeⅡ (AT-Ⅱ) cells and bronchiolar epithelial and is prominently expressed when AT-Ⅱcells were damaged or regenerated [35]. Serum KL-6 is significantly correlated with computed tomography score and can help us to assess disease severity in COVID-19, which might be because SARS-CoV-2 mainly damages AT-Ⅱ cells through ACE2 [36, 37, 38, 39]. However, in our study, RAGE was better than KL-6 in terms of prediction of ARDS. As a biomarker of lung epithelium injury, expression of RAGE is significantly upregulated in the lung epithelium, especially in alveolar typeⅠ (AT-Ⅰ) cells [35]. AT-Ⅰcells participate in the formation of the blood–air barrier and are relatively more vulnerable to injury. AT-Ⅱ cells secrete alveolar surface active material and can be converted into AT-Ⅰcells, but they do not participate in the blood–air barrier and are more tolerant to damage. Therefore, for ARDS, RAGE, as a marker of AT-Ⅰcells injury, is more predictive than KL-6, which is a marker of AT-Ⅱ cells injury.

We also analyzed IFN-*γ*, TNF-*α*, MCP-1, IL-6, and IL-10 representative of different pathophysiological disease-related changes during ARDS development. In our study, after univariate and multivariate analysis, except for IL-8 and RAGE, the rest of the plasma biomarkers were all excluded. A review indicated that IFN-*γ* and TNF-*α* were associated with the prognosis of disease [40], but our aim was to predict the occurrence of ARDS, which might be the reason why differences between IFN-*γ* and TNF-*α* were not remarkable between two groups, and it was proved that the concentration of MCP-1 in ventilator-associated pneumonia patients with ARDS was significantly higher than that in patients without ARDS (*p*=0.04), but the blood plasma samples in study we mentioned above were collected after the occurrence of ARDS, so the results were slightly different from ours [41]. IL-6, IL-8, and IL-10 showed differences between two groups on the first day in our study, but only IL-8 was included in our predictive model, which might be because levels of IL-6 and IL-10 had correlations with IL-8, the correlation coefficient of which are shown in Table S5 and Figure S1. In conclusion, due to the differences in research designs and time of plasma collection, the inflammatory mediators and cytokines summarized in different studies vary greatly. In some previous studies, the enrolled patients were already diagnosed with ARDS, and the specific onset time was unknown. However, in our current study, patients with high-risk factors for ARDS who had not yet developed ARDS were enrolled. By closely monitoring the patients' condition until the seventh day (non-ARDS) or the day after the onset of ARDS, we were able to obtain a clear date of ARDS onset. The biomarkers obtained prior to this time point are, therefore, more predictive in value.

As we mentioned above, differences in levels of plasma biomarkers on the second (third or fourth) day between ARDS and non-ARDS patients were not significant, that might be because we applied bundle therapies for patients according to international guidelines for corresponding diseases, among which, the removal of predisposition, antishock, antimicrobial therapy, stress reduction, and others might partially terminate further damage to the lung by harmful factors. Meanwhile, antishock therapy and fluid resuscitation might dilute the concentration of plasma biomarkers. In other words, the subsequent variation in plasma biomarkers did not occur as expected, which might be related to therapeutic intervention. After repeated statistical comparison, we considered that the difference of plasma biomarkers between ARDS and non-ARDS patients on the first day was the most significant and had the most predictive value.

In terms of clinical scoring, LIPS, constructed by Gajic et al. [42], has been proven that it could alert clinicians about the risk of ALI and facilitate testing and implementation of ALI prevention strategies. However, its usefulness is limited due to its relatively low positive predictive value [43]. Although the performance of LIPS is inconsistent in different countries, it was widely recognized due to its large samples size and external verification. So in our study, we still used it as an independent variable of the model, which can increase the authority and consensus of it.

PaO_2_/FiO_2_ is considered one of the essential indicators in the diagnosis of ARDS. Douville et al. [44] found that higher postoperative PaO_2_/FiO_2_ was associated with a reduced risk of pulmonary complications, and lower postoperative PaO_2_/FiO_2_ was independently associated with pulmonary complications and mortality [44, 45, 46]. Previous studies prompted us that biomarkers were helpful in assisting clinical identification and mortality prediction of ARDS based on PaO_2_/FiO_2_ [13]. Indeed, our model was superior to PaO_2_/FiO_2_ alone for predicting ARDS, which was supported by NRI (NRI: 0.335^*∗∗*^,95% CI: 0.003–0.411).

There were only a few studies predicting the occurrence of ARDS, among which, most of them only targeted a certain high-risk group, such as sepsis, pancreatitis, COVID-19, and so on, while our study established a model of predictive value for all ARDS high-risk patients in SICU. In the few studies involving plasma biomarkers, for overall patients, a prospective study enrolled 158 Han Chinese patients with ARDS risk factors from the respiratory and emergency intensive care units showed that AUC of LIPS + AngⅡ for predicting occurrence of ARDS was 0.803 (95% CI: 0.727−0.879), sensitivity and specificity were 0.711 and 0.797, respectively [43], LIPS + AngⅡ also exhibits good predictive capability in our population, with an AUC of 0.814 (95% CI: 0.760−0.869), a sensitivity of 0.853, but a decreased specificity of 0.697. Nevertheless, our model still demonstrates certain advantages, it applied in high-risk patients of SICU was 0.883 (95% CI: 0.835−0.931, sensitivity 0.933, specificity 0.730). For sepsis patients, the AUC of our model was 0.844 (95% CI: 0.764−0.924). We also attempted to use KL-6 alone to predict ARDS, but the results were poor, with an AUC of 0.523 (95% CI: 0.438–0.608) and sensitivity and specificity of 0.567 and 0.429, respectively. In addition, in a prospective study enrolled of 232 sepsis patients, the AUC of combination of PaO_2_/FiO_2_, RAGE, SP-D, AngⅡ and CXCL16 was 0.881 (95% CI: 0.837−0.925) [47], and in Lorraine's research, AUC of multivariable model (includes SPD, RAGE, IL-8, CC16, IL-6) was 0.750 (95% CI: 0.700−0.840) [28]. In our study, we did not measure factors like SP-D, CXCL16, and CC16, thus, we were unable to fully observe the performance of these two previous models in our cohort. Therefore, despite the prediction models mentioned above, the performances were varied and may be partly related to the different patient populations.

In brief, our study is prospective that conducted in a high-risk population before the onset of ARDS, differing from previous research designs, and thus, the conclusions do not fully align with those of previous studies.

Although strict inclusion and exclusion criteria were used in the present study to establish a better prediction model than clinical parameters or plasma biomarkers alone, our study had several limitations: (1) The sample size was small, and we only measured nine plasma biomarkers as representative, there also exist a variety of other biomarkers. We need more samples and multicenter studies to expand and verify our conclusions. (2) We did not compare plasma biomarkers with bronchoalveolar lavage fluid biomarkers, which may limited our understanding of some biomarkers related to epithelial injury. However, designing such a study would be ethically challenging and difficult for patients to accept. (3) We did not make the best use of our data based on time series. We need more samples to attain the variation tendency of biomarkers by trajectory analysis. However, as mentioned above, due to the use of active and effective treatment, there were no statistical differences in plasma biomarkers between the two groups from the second day onwards, making it difficult to achieve the desired results even with trajectory analysis. Nevertheless, there were some strengths in our study: (1) We collected samples consecutively before the onset of ARDS, which made our result of prediction more reliable. (2) We established the prediction model of ARDS in SICU based on the currently recognized LIPS, thus significantly improved the practicability of our model. (3) We combined plasma biomarkers and clinical parameters, attempting to build a foundation of a more comprehensive predictive model for ARDS.

## 5. Conclusions

In this prospective study cohort, we found an association between clinical parameters, plasma biomarkers, and the onset of ARDS among patients with high risks of ARDS. LIPS, PaO_2_/FiO_2_, IL-8, and RAGE of the first day were identified as independent risk factors for ARDS. The predictive model established based on their combination showed significant predictive value for the occurrence of ARDS in the SICU.

## Figures and Tables

**Table 1:** Baseline characteristics and clinical/laboratory parameters.

Variable	All patients (*n* = 263)	ARDS (*n* = 84)	Non-ARDS (*n* = 179)	*p*-Value
Age (year) (Q1–Q3)	62 (49–73)	66.0 (53.5–75.8)	60 (46.0–71.0)	0.012^*∗*^
Sex (male) (%)	107 (40.7%)	22 (26.1%)	85 (47.5%)	0.001^*∗∗*^
BMI (Q1–Q3)	24.5 (22.0–27.3)	24.2 (22.7–27.3)	24.7 (21.8–27.3)	0.996
Predisposing conditions for ARDS				
Shock (%)	110 (41.8%)	51 (60.7%)	59 (33.0%)	<0.001^*∗∗*^
Multiple trauma (%)	44 (16.7)	15 (17.9%)	29 (16.2%)	0.737
Traumatic brain injury (%)	54 (20.5)	17 (20.2%)	37 (20.7%)	0.936
Sepsis (%)	104 (39.5%)	41 (48.8%)	63 (35.2%)	0.035^*∗*^
Acute abdomen (%)	110 (41.8)	47 (56.0%)	59 (33.0%)	0.001^*∗∗*^
Pulmonary contusion (%)	18 (6.8%)	11 (13.1%)	7 (3.9%)	0.016^*∗*^
Pneumonia (%)	34 (12.9%)	19 (22.6%)	15 (8.4%)	0.001^*∗∗*^
High-risk parturients (%)	12 (4.6%)	1 (1.2%)	11 (6.2%)	0.073
Aspiration (%)	7 (2.7%)	7 (8.3%)	0 (0.0%)	<0.001^*∗∗*^
Spinal corrective surgery (%)	29 (11.0%)	4 (4.8%)	25 (14.0)	0.026^*∗*^
Cervical spinal cord injury (%)	43 (16.3%)	9 (10.7%)	34 (19.0)	0.090
Comorbidity				
Chronic obstructive pulmonary disease (%)	3 (1.1%)	0 (0.0%)	3 (1.7%)	0.233
Hypertension (%)	126 (47.9%)	46 (54.8%)	80 (44.7%)	0.128
Coronary heart disease (%)	31 (11.8%)	11 (13.1%)	20 (11.2%)	0.652
Diabetes (%)	62 (23.5%)	25 (29.8%)	37 (20.7%)	0.105
Hepatic dysfunction (%)	51 (19.4%)	23 (27.4%)	28 (15.6%)	0.025^*∗*^
Chronic kidney disease (%)	14 (5.3%)	7 (8.3%)	7 (3.9%)	0.136
Malignant tumor (%)	44 (16.7%)	16 (19.0%)	28 (15.6%)	0.490
APACHEⅡ score (Q1–Q3)	17 (13–20)	18.0 (14.0–22.0)	17 (13.0–19.0)	0.003^*∗∗*^
LIPS (Q1–Q3)	5.5 (4–8)	8.0 (6.5–10.0)	5.0 (3.5–6.5)	<0.001^*∗∗*^
SOFA score (Q1–Q3)	7 (5–9)	9.0 (7.0–10.0)	7.0 (5.0–9.0)	<0.001^*∗∗*^
PaO_2_/FiO_2_ (Q1–Q3)	254.0 (183–342)	183.0 (135.3–216.0)	300 (222.5–370.0)	<0.001^*∗∗*^
Use of vasopressors (%)	191 (72.6%)	68 (81.0%)	123 (68.7%)	0.038^*∗*^
Use of CRRT (%)	59 (22.4%)	23 (27.4%)	36 (20.1%)	0.188
Invasive mechanical ventilation (%)	229 (87.1%)	77 (91.7%)	152 (84.9%)	0.128
Days on IMV (Q1–Q3)	7 (3–13)	9.5 (6–16)	6 (3–11)	<0.001^*∗∗*^
SICU days (Q1–Q3)	11 (6–19)	13.5 (8–23)	10 (5–17)	0.002^*∗∗*^

Significant at  ^*∗*^*p*  < 0.05, ^*∗∗*^*p*  < 0.01. BMI, body mass index; APACHEⅡ, acute physiology and chronic health evaluation Ⅱ; LIPS, lung injury prediction score; SOFA, sequential organ failure assessment; CRRT, continuous renal replacement therapy; IMV, invasive mechanical ventilation; SICU, surgical intensive care unit; Q1, quartiles 1; Q3, quartiles 3.

**Table 2:** Univariate and multivariate analysis.

Variables	Univariate HR (95% CI)	*p*-Value	Multivariate HR (95 % CI)	*p*-Value
IL-6_D1	1.438 (1.111–1.862)	0.006	—	—
IL-8_D1	1.579 (1.074–2.322)	0.02	1.657 (1.087–2.526)	0.019
AngⅡ_D1	2.054 (1.167–3.614)	0.015	—	—
RAGE_D1	2.918 (0.783–10.865)	0.11	4.252 (1.251–14.452)	0.02
IL-6/IL-10_D1	1.297 (0.949–1.771)	0.103	—	—
LIPS	1.349 (1.243–1.463)	<0.001	1.290 (1.171–1.421)	<0.001
APACHEⅡ	1.077 (1.032–1.124)	<0.001	—	—
SOFA	1.109 (1.036–1.188)	0.003	—	—
PaO_2_/FiO_2_	0.989 (0.986–0.992)	<0.001	0.992 (0.989–0.995)	<0.001
Age	1.019 (1.004–1.033)	0.011	—	—
Sex	0.371 (0.214–0.645)	<0.001	—	—
Shock	2.623 (1.642–4.191)	<0.001	—	—
Sepsis	1.768 (1.123–2.783)	0.014	—	—
Acute abdomen	2.121 (1.339–3.360)	0.001	—	—
Pulmonary contusion	1.834 (1.232–2.730)	0.003	—	—
Pneumonia	2.354 (1.353–4.096)	0.002	—	—
Spinal corrective surgery	0.412 (0.150–1.128)	0.084	—	—
Hypertension	1.433 (0.908–2.261)	0.122	—	—
Diabetes	1.439 (0.875–2.366)	0.151	—	—
Hepatic dysfunction	1.863 (1.133–3.064)	0.014	—	—

HR, hazard ratio; 95% CI, 95% confidence interval; IL-6, interleukin-6; IL-8, interleukin-8; AngⅡ, angiopoietin Ⅱ; RAGE, receptor for advanced glycation end-products; LIPS, lung injury prediction score; APACHEⅡ, acute physiology and chronic health evaluation Ⅱ; SOFA, sequential organ failure assessment.

**Table 3:** C-index of predicting the occurrence of ARDS on different days.

	C-index	95% CI	SE
All patients	0.864	0.828–0.900	0.019
Predict onset of ARDS on the second day	0.937	0.899–0.975	0.019
Predict onset of ARDS on the third day	0.923	0.869–0.977	0.028
Predict onset of ARDS after the third day	0.898	0.850–0.946	0.025

95% CI, 95% confidence interval; SE, standard error.

**Table 4:** Results of fivefold cross-validation.

	Minimum	Q1	Median	Mean	Q3	Maximum
Train cohort	0.761	0.84	0.865	0.858	0.883	0.937
Validation cohort	0.849	0.86	0.865	0.867	0.872	0.887

Q1, quartiles 1; Q3, quartiles 3.

## Data Availability

The datasets used during the current study are available from the corresponding author upon reasonable request.

## Supplementary Materials

Supplementary Materials Table S1: levels of plasma biomarkers on the first day between ARDS and Non-ARDS patients; Table S2: levels of plasma biomarkers on the second (third or fourth) day between ARDS and Non-ARDS patients; Table S3: collinearity analysis of independent risk factors; Table S4: C-index of IL-8,RAGE,LIPS or PaO_2_/FiO_2_ alone and predictive value of the model compared with them; Table S5: Pearson correlation between IL-8 and IL-6 or IL-10; Figure S1: Scatter plot between IL-8 and IL-10 or IL-6.
