# Supplementary material for: LIPS and PaO2/FiO2 Combined Plasma Biomarkers Predict Onset of Acute Respiratory Distress Syndrome in Patients of High Risks in SICU: A Prospective Exploratory Study
Source: Mediators Inflamm. 2024 Sep 17;2024:4936265. doi: 10.1155/2024/4936265 (PMC11421942; doi:10.1155/2024/4936265)
Supplement: Supplementary Materials — Table S1: levels of plasma biomarkers on the first day between ARDS and Non-ARDS patients; Table S2: levels of plasma biomarkers on the second (third or fourth) day between ARDS and Non-ARDS patients; Table S3: collinearity analysis of independent risk factors; Table S4: C-index of IL-8,RAGE,LIPS or PaO2/FiO2 alone and predictive value of the model compared with them; Table S5: Pearson correlation between IL-8 and IL-6 or IL-10; Figure S1: Scatter plot between IL-8 and IL-10 or IL-6. [file 4936265.f1.doc]

**Supplementary Table 1:Levels of plasma biomarkers on the first day between ARDS and Non-ARDS patients**

a.All patients b.Extrapulmonary patients

| Variable | Non-ARDS | ARDS | P value |
| --- | --- | --- | --- |
| IFN-γ_D1 | 1.05±0.61 | 1.00±0.65 | 0.558 |
| TNF-α_D1 | 1.42±0.53 | 1.49±0.52 | 0.343 |
| MCP-1_D1 | 2.49±0.52 | 2.59±0.49 | 0.191 |
| IL-6 _D1 | 2.35±0.80 | 2.69±0.87 | 0.004** |
| IL-8_D1 | 2.01±0.60 | 2.20±0.54 | 0.018* |
| IL-10_D1 | 1.52±0.72 | 1.64±0.72 | 0.235 |
| AngⅡ_D1 | 3.31±0.34 | 3.43±0.47 | 0.047* |
| KL-6_D1 | 1.48±0.20 | 1.52±0.25 | 0.233 |
| RAGE_D1 | 2.44±0.15 | 2.48±0.22 | 0.177 |
| IL-6/IL-10_D1 | 0.89±0.69 | 1.07±0.79 | 0.068 |

| Variable | Non-ARDS | ARDS | P value |
| --- | --- | --- | --- |
| IFN-γ_D1 | 1.06±0.61 | 1.03±0.59 | 0.745 |
| TNF-α_D1 | 1.41±0.54 | 1.50±0.47 | 0.248 |
| MCP-1_D1 | 2.51±0.52 | 2.62±0.53 | 0.209 |
| IL-6 _D1 | 2.36±0.81 | 2.78±0.89 | 0.002** |
| IL-8_D1 | 2.02±0.60 | 2.24±0.52 | 0.022* |
| IL-10_D1 | 1.52±0.73 | 1.67±0.72 | 0.176 |
| AngⅡ_D1 | 3.32±0.34 | 3.49±0.48 | 0.018* |
| KL-6_D1 | 1.49±0.19 | 1.49±0.23 | 0.82 |
| RAGE_D1 | 2.45±0.15 | 2.48±0.23 | 0.31 |
| IL-6/IL-10_D1 | 0.90±0.69 | 1.13±0.80 | 0.039* |

c.Severe and moderate patients

| Variable | Non-ARDS | ARDS | P value |
| --- | --- | --- | --- |
| IFN-γ_D1 | 1.05±0.61 | 1.04±0.68 | 0.878 |
| TNF-α_D1 | 1.42±0.53 | 1.52±0.53 | 0.216 |
| MCP-1_D1 | 2.49±0.52 | 2.59±0.48 | 0.225 |
| IL-6 _D1 | 2.35±0.80 | 2.83±0.89 | <0.001** |
| IL-8_D1 | 2.01±0.60 | 2.25±0.53 | 0.009** |
| IL-10_D1 | 1.52±0.72 | 1.76±0.68 | 0.031* |
| AngⅡ_D1 | 3.31±0.34 | 3.47±0.49 | 0.027* |
| KL-6_D1 | 1.48±0.20 | 1.55±0.24 | 0.051 |
| RAGE_D1 | 2.44±0.15 | 2.48±0.21 | 0.237 |
| IL-6/IL-10_D1 | 0.89±0.69 | 1.06±0.74 | 0.109 |

Significant at *p<0.05,**p<0.01.IFN-γ:interferon-γ;TNF-α:tumor necrosis factor-α;MCP-1:monocyte chemotactic protein-1;IL-6:interleukin-6;IL-8:interleukin-8;IL-10:interleukin-10;AngⅡ:angiopoietinⅡ;KL-6:krebs von den Lungen-6;RAGE:receptor for advanced glycation end-products

**Supplementary Table 2:Levels of plasma biomarkers on the second(third or fourth) day between ARDS and Non-ARDS patients**

a.Day2 b.Day3

| Variable | ARDS | Non-ARDS | P value |
| --- | --- | --- | --- |
| IFN-γ_D2 | 0.92±0.47 | 0.94±0.75 | 0.838 |
| TNF-α_D2 | 1.32±0.49 | 1.34±0.73 | 0.847 |
| MCP-1_D2 | 2.42±0.53 | 2.42±0.51 | 0.982 |
| IL-6 _D2 | 2.24±0.78 | 2.17±0.74 | 0.583 |
| IL-8_D2 | 1.93±0.68 | 1.91±0.68 | 0.799 |
| IL-10_D2 | 1.18±0.87 | 1.31±0.87 | 0.349 |
| AngⅡ_D2 | 3.32±0.43 | 3.38±0.45 | 0.416 |
| KL-6_D2 | 1.56±0.22 | 1.50±0.27 | 0.182 |
| RAGE_D2 | 2.46±0.19 | 2.52±0.28 | 0.134 |
| IL-6/IL-10_D2 | 1.06±0.94 | 0.86±0.78 | 0.133 |

| Variable | ARDS | Non-ARDS | P value |
| --- | --- | --- | --- |
| IFN-γ_D3 | 0.81±0.64 | 0.93±0.84 | 0.547 |
| TNF-α_D3 | 1.30±0.54 | 1.33±0.79 | 0.852 |
| MCP-1_D3 | 2.37±0.43 | 2.36±0.48 | 0.96 |
| IL-6 _D3 | 1.91±0.63 | 1.96±0.71 | 0.743 |
| IL-8_D3 | 1.87±0.54 | 1.84±0.70 | 0.84 |
| IL-10_D3 | 0.98±1.06 | 1.29±0.83 | 0.114 |
| AngⅡ_D3 | 3.25±0.32 | 3.31±0.37 | 0.488 |
| KL-6_D3 | 1.56±0.23 | 1.52±0.26 | 0.488 |
| RAGE_D3 | 2.44±0.16 | 2.50±0.27 | 0.289 |
| IL-6/IL-10_D3 | 0.94±0.92 | 0.67±0.72 | 0.129 |

c.Day4

| Variable | ARDS | Non-ARDS | P value |
| --- | --- | --- | --- |
| IFN-γ_D4 | 1.07±0.70 | 1.05±0.80 | 0.928 |
| TNF-α_D4 | 1.45±0.64 | 1.40±0.80 | 0.867 |
| MCP-1_D4 | 2.44±0.45 | 2.31±0.45 | 0.432 |
| IL-6 _D4 | 2.16±0.61 | 1.92±0.66 | 0.318 |
| IL-8_D4 | 2.02±0.44 | 1.90±0.65 | 0.595 |
| IL-10_D4 | 1.19±0.95 | 1.32±0.82 | 0.665 |
| AngⅡ_D4 | 3.31±0.36 | 3.28±0.42 | 0.797 |
| KL-6_D4 | 1.53±0.16 | 1.56±0.28 | 0.817 |
| RAGE_D4 | 2.45±0.10 | 2.48±0.27 | 0.811 |
| IL-6/IL-10_D4 | 0.97±0.80 | 0.60±0.65 | 0.126 |

Significant at *p<0.05,**p<0.01.IFN-γ:interferon-γ;TNF-α:tumor necrosis factor-α;MCP-1:monocyte chemotactic protein-1;IL-6:interleukin-6;IL-8:interleukin-8;IL-10:interleukin-10;AngⅡ:angiopoietinⅡ;KL-6:krebs von den Lungen-6;RAGE:receptor for advanced glycation end-products

# **Supplementary Table 3:Collinearity analysis of independent risk factors**

|  | VIF | Tolerance |
| --- | --- | --- |
| IL-8_D1 | 1.119 | 0.893 |
| RAGE_D1 | 1.036 | 0.965 |
| LIPS score | 1.218 | 0.821 |
| PaO_2_/FiO_2_ | 1.131 | 0.884 |

IL-8:interleukin-8;RAGE:receptor for advanced glycation end-products;LIPS:lung injury prediction score;VIF:variance inflation factor

**Supplementary Table 4:C-index of IL-8,RAGE,LIPS or PaO_2_/FiO_2_ alone and predictive value of the model compared with them**

|  | C-index | 95%CI | SE | NRI | 95%Cl |
| --- | --- | --- | --- | --- | --- |
| LIPS | 0.781 | 0.729-0.834 | 0.027 | 0.462** | 0.093-0.526 |
| PaO_2_/FiO_2_ | 0.799 | 0.751-0.846 | 0.024 | 0.335** | 0.003-0.411 |
| IL-8_D1 | 0.605 | 0.532-0.677 | 0.037 | 0.597** | 0.098-0.698 |
| RAGE_D1 | 0.537 | 0.461-0.612 | 0.038 | 0.596** | 0.086-0.668 |

Significant at *p<0.05,**p<0.01.95%CI:95%confidence interval;SE:standard error;NRI:net reclassification index

**Supplementary Table 5:Pearson correlation between IL-8 and IL-6 or IL-10**

|  | IL-8_D1 | IL-10_D1 | IL-6 _D1 |
| --- | --- | --- | --- |
| IL-8_D1 | 1 |  |  |
| IL-10_D1 | 0.529** | 1 |  |
| IL-6 _D1 | 0.561** | 0.610** | 1 |

Significant at *p<0.05,**p<0.01.IL-8:interleukin-8;IL-10:interleukin-10;IL-6:interleukin-6


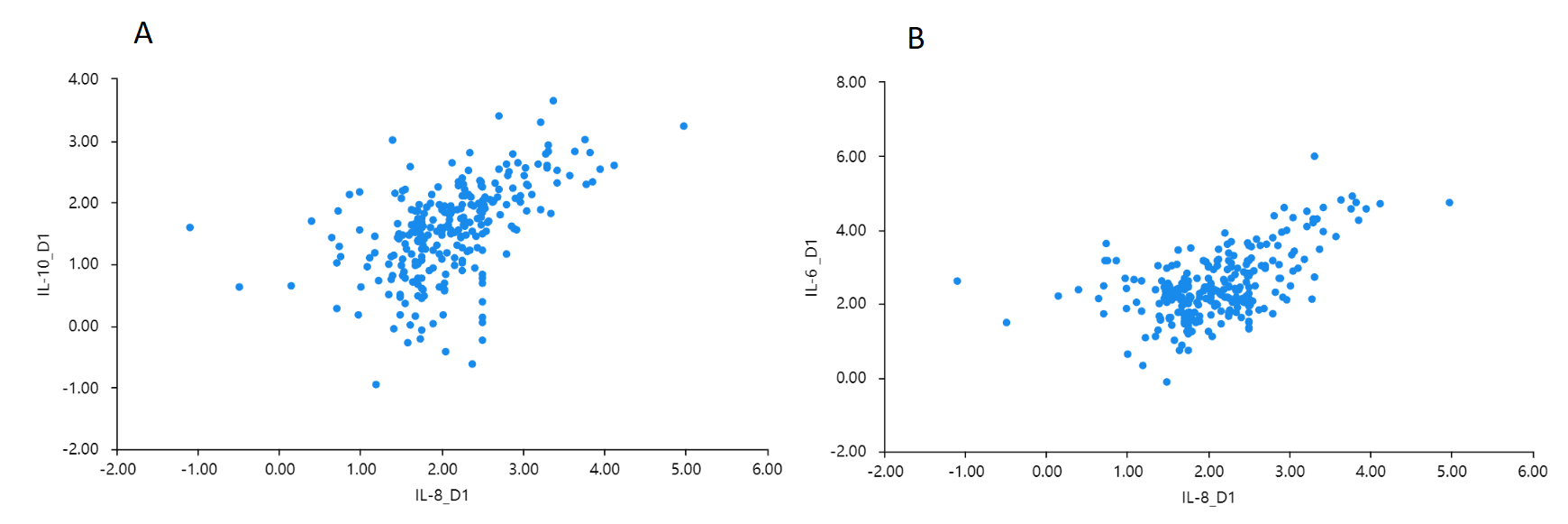


**Supplementary Figure 1:Scatter plot between IL-8 and IL-10 or IL-6.IL-8:interleukin-8;IL-10:interleukin-10;IL-6:interleukin-6**
